# Prolonged Post-Harvest Preservation in Lettuce (*Lactuca sativa* L.) by Reducing Water Loss Rate and Chlorophyll Degradation Regulated through Lighting Direction-Induced Morphophysiological Improvements

**DOI:** 10.3390/plants13182564

**Published:** 2024-09-12

**Authors:** Jingli Yang, Jinnan Song, Jie Liu, Xinxiu Dong, Haijun Zhang, Byoung Ryong Jeong

**Affiliations:** 1 Weifang Key Laboratory for Stress Resistance and High Yield Regulation of Horticultural Crops, Shandong Provincial University Laboratory for Protected Horticulture, College of Jia Sixie Agriculture, Weifang University of Science and Technology, Shouguang 262700, China or yangmiaomiaode@gmail.com (J.Y.); jinnansong93@gmail.com (J.S.); liujie655@163.com (J.L.); dongxinxiu2022@163.com (X.D.); haijunz627@163.com (H.Z.); 2 Department of Horticulture, Division of Applied Life Science (BK21 Four), Graduate School, Gyeongsang National University, Jinju 52828, Republic of Korea; 3 Division of Horticultural Science, College of Agriculture and Life Sciences, Gyeongsang National University, Jinju 52828, Republic of Korea

**Keywords:** chlorophyll degradation, cultivation light direction, leaf vegetable, post-harvest storage senescence, relative conductivity, water loss rate

## Abstract

To investigate the relationship between the lighting direction-induced morphophysiological traits and post-harvest storage of lettuce, the effects of different lighting directions (top, T; top + side, TS; top + bottom, TB; side + bottom, SB; and top + side + bottom, TSB; the light from different directions for a sum of light intensity of 600 μmol·m^−2^·s^−1^ photosynthetic photon flux density (PPFD)) on the growth morphology, root development, leaf thickness, stomatal density, chlorophyll concentration, photosynthesis, and chlorophyll fluorescence, as well as the content of nutrition such as carbohydrates and soluble proteins in lettuce were analyzed. Subsequently, the changes in water loss rate, membrane permeability (measured as relative conductivity and malondialdehyde (MDA) content), brittleness (assessed by both brittleness index and β-galactosidase (β-GAL) activity), and yellowing degree (evaluated based on chlorophyll content, and activities of chlorophyllase (CLH) and pheophytinase (PPH)) were investigated during the storage after harvest. The findings indicate that the TS treatment can effectively reduce shoot height, increase crown width, enhance leaves’ length, width, number, and thickness, and improve chlorophyll fluorescence characteristics, photosynthetic capacity, and nutrient content in lettuce before harvest. Specifically, lettuce’s leaf thickness and stomatal density showed a significant increase. Reasonable regulation of water loss in post-harvested lettuce is essential for delaying chlorophyll degradation. It was utilized to mitigate the increase in conductivity and hinder the accumulation of MDA in lettuce. The softening speed of leafy vegetables was delayed by effectively regulating the activity of the β-GAL. Chlorophyll degradation was alleviated by affecting CLH and PPH activities. This provides a theoretical basis for investigating the relationship between creating a favorable light environment and enhancing the post-harvest preservation of leafy vegetables, thus prolonging their post-harvest storage period through optimization of their morphophysiological phenotypes.

## 1. Introduction

Light plays a crucial role as an environmental cue, significantly impacting every stage of a plant’s life cycle. The growth and development of plants are influenced by both the quality and quantity of light. As is well known, light serves as a complex signaling input that influences the morphophysiology of plants. Furthermore, plants require multiple photoreceptors to correctly sense and process light input [1,2]. Leaves typically have a large surface to maximize their absorption of sunlight [3].

Variations in the lighting direction have an impact on plant morphophysiology. The interception of light is directly affected by the leaf orientation. Our previous study revealed that optimizing the positioning of lighting, adjusting for favorable lighting direction, and controlling the maximum leaf area exposed to light can significantly improve plant performance [4]. This enhancement is achieved through increased photosynthesis, which results in a higher light use efficiency [5,6]. Under light competition conditions, phototropism-induced changes in leaf angle and movement (epinastic or hyponastic) are thought to improve photosynthetic capacity, efficiency, and carbon gain [7,8,9]. The leaf angle changes stimulate the midrib epidermal cells, which affects the stomatal status. Stomatal density and size are essential factors reflecting plants’ ability to adapt to environmental changes [10,11]. The most effective strategy to achieve higher stomatal conductance at low CO_2_ levels, which enhances photosynthetic efficiency, is to have denser, more open, and smaller stomata [12].

Leafy vegetables are rich in carbohydrates, vitamins, minerals, and other nutrients and are popular in people’s daily lives. However, the water loss of leafy vegetables is rapid after harvest due to their large leaves, high water content, and brittle tissues. This makes them susceptible to mechanical damage. After harvest, leaf membrane damage affected conductivity and the content of malondialdehyde (MDA). At the same time, plant wounds lead to leaf wilting, accelerate leaf softening, and increase β-galactosidase (β-GAL) activity [13]. Furthermore, the activities of pheophytinase (PPH) and chlorophyllase (CLH) affect the degradation of chlorophyll [14], resulting in the discoloration and yellowing of leafy vegetables. Therefore, leafy vegetables have become the most challenging agricultural products to keep fresh [15]. Hence, targeted post-harvest storage technology can significantly reduce post-harvest loss and improve quality.

Lettuce (*Lactuca sativa* L.) is a biennial herb with large green leaves and high nutritional value that are well-suited for consumption in their raw state. After being harvested, lettuce is susceptible to water loss, wilting, and browning due to its respiration and transpiration. At the same time, its brittleness and high water content make it susceptible to damage during harvesting, transportation, and storage, further hastening its deterioration of quality [16].

Numerous studies have been conducted on the preservation of leaf vegetables. Yu Jiangtao et al. reviewed the methods of lettuce fresh-keeping, including physical methods (vacuum pre-cooling, packaging film and air conditioning, low temperature, heat treatment, light, irradiation, and high pressure), chemical methods (disinfectant cleaning and chemical preservatives), and biological methods (biocides and natural extracts), etc. [17]. The preservation of leafy vegetables commonly depends on the application of these technologies [18,19]. Jiang Wenli et al. studied the effects of low-temperature humidification on the storage and fresh-keeping of lettuce [20]. Additionally, other researchers investigated the impact of different packaging materials on the physiology of lettuce and its fresh-keeping quality in the post-harvest duration [21,22]. The mechanism of the fresh-keeping method for leafy vegetables remained unknown until Li Xuerui et al. [23] discovered that 1-Methylcyclopropene (1-MCP) combined with low temperatures could regulate brittleness and chlorophyll degradation, thereby alleviating post-harvest senescence of leafy vegetables. This provides a theoretical basis for investigating the preservation mechanism of leafy vegetables and further utilizing post-harvest technology to extend their storage period. Moreover, the most closely related to our study is a report by Min et al., who concluded that high light intensity applied shortly before harvest improves lettuce’s nutritional quality and extends its shelf life [24]. The use of specific light treatment before harvest also affects the storage period of lettuce by changing its physiological characteristics. Through their study, the levels of ascorbic acid (AsA) and carbohydrates at harvest correlated positively with the subsequent shelf life, indicating that the prolonged shelf life relies on the improved energy and antioxidant status of the crop at harvest.

The purpose and significance of this study are outlined in the following three aspects: (1) Highlighted the regulation of “light direction” on plant morphophysiology in protected horticulture. (2) Explored the optimum combination of light directions, which can produce the best growth state, the highest nutrient content, and the highest light utilization efficiency, and enhanced the application potential of indoor leaf vegetable cultivation in plant factories. (3) Investigated how to reduce the post-harvest storage aging of lettuce by studying various physiological indicators (such as water loss rate, relative conductivity, MDA content, brittleness, β-GAL activity, and chlorophyll degradation rate) of different lighting direction-cultivated lettuces. The main focus of this study is to extend the post-harvest storage of lettuce by improving its growth and quality. This storage process does not require special storage conditions, special packaging, chemical preservatives, sterilization, disinfectants, etc. This innovative light cultivation provides a new idea to prolong the freshness of post-harvested lettuce and improves the green, healthy, and environmental quality of lettuce. Moreover, the lower economic cost, low food safety risk, and low ecological pressure of this lighting cultivation also lay a good foundation for its future promotion and application.

## 2. Results

### 2.1. Morphological and Growth Parameters

**Figure 1:**
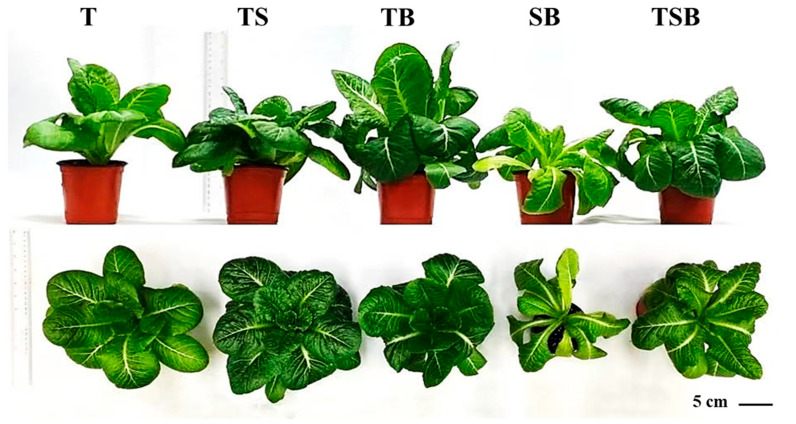
The morphology of lettuce (*Lactuca sativa* L.) ‘Caesar Green’ plants under various lighting directions for 45 days. The bar indicates 5 cm.

**Figure 2:**
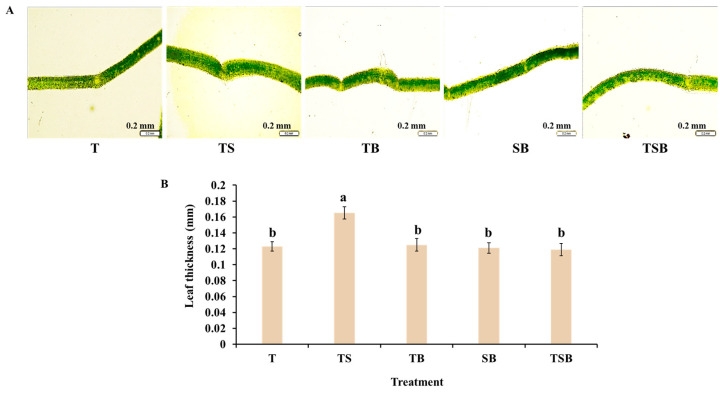
The thickness of lettuce leaves under various lighting directions during 45 days of cultivation. (**A**) The micrograph of the cross-section of the middle section of the mature fourth leaf from the top; the bars indicate 0.2 mm. (**B**) The leaf thickness at the same site under different treatments. Vertical bars represent means ± standard error (n = 9). Different lowercase letters indicate significant differences within treatments by Duncan’s multiple range test at *p* ≤ 0.05.

After 45 days of cultivation, different lighting direction combinations significantly impacted the plants’ growth and morphological parameters. In this study, the stems and leaves of lettuce in TS treatment were uniformly spread to the upper oblique, with flourishing growth and many stretched leaves. The degree of occlusion between leaves was small, and a larger light area could be obtained under the same circumstances. Although lettuces in TS did not achieve the highest shoot height, second only to the TB and T treatments, it resulted in the largest crown width, fresh weight, and dry weight, as well as the most leaf number (Figure 1 and Table 1). As shown in Figure 1, due to the positive phototropism of stems and leaves in the aerial parts of higher plants, different light directions led to significant differences in lettuce phenotypes among treatments at harvest. Lettuces irradiated by different light directions caused various degrees of growth bending of stems and leaves, which eventually led to differences in shoot height (side view) and crown width (top view) (Figure 1). In the SB treatment, lettuces were only exposed to light from the side and bottom, and their stems and leaves extended and drooped to the side and below to the greatest extent, resulting in the curly and slender leaves so that the shoot height and crown width in this treatment were the lowest. In addition, due to the large number of light receptors on the adaxial leaves of green plants, the unreasonable light direction in the SB treatment was not conducive to lettuces obtaining more light, which was not conducive to photosynthesis. Compared with other light treatments, the SB treatment cannot maintain better growth of lettuces, so the minimum shoot fresh weight and dry weight also appeared in this treatment (Figure 1 and Table 1). Additionally, although different treatments led to significant differences in the number, length, and width of lettuce leaves, only the TS treatment significantly thickened the leaves, and there was no difference in leaf thickness among the other treatments (Figure 2).

**Figure 3:**
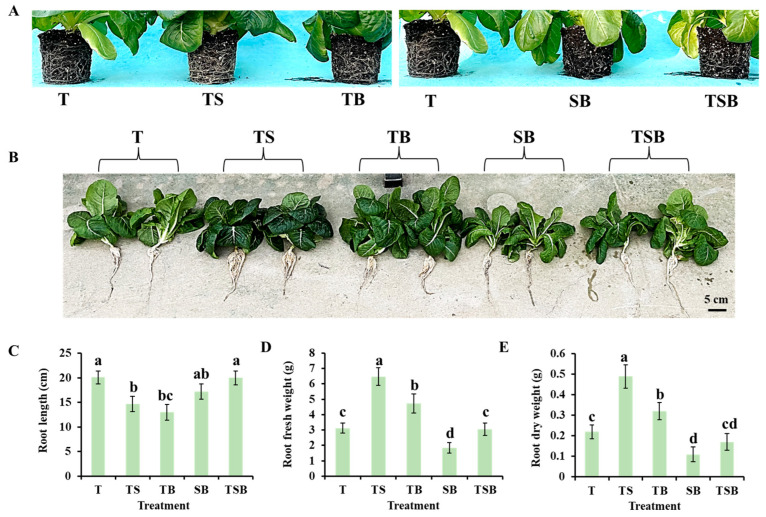
The growth and development of lettuce roots under various lighting directions during 45 days of cultivation. (**A**) The raw state of the lettuce root after removing the pot. (**B**) The state of the cleaned lettuce roots; the bar indicates 5 cm. (**C**) Root length (average most extended root length). (**D**) Root fresh weight. (**E**) Root dry weight. Vertical bars represent means ± standard error (n = 9). Different lowercase letters indicate significant differences within treatments by Duncan’s multiple range test at *p* ≤ 0.05.

After 45 days of cultivation, at harvest, from the raw state of roots after pot removal, the lettuce in the TS treatment had a more developed root system (Figure 3A), formed a “root-soil” complex, and had a more vital ability to wrap and stabilize soil, which was of great significance for preventing soil erosion and improving soil water holding capacity. At the same time, the well-developed root system of the plant can extend to deeper soil layers to obtain more water and nutrition. According to the cleaned root status of lettuces, the TS treatment also formed the most developed root system, although not the longest, but the root with the largest fresh and dry weight (Figure 3B,C). In contrast, the lettuce treated with the SB treatment had the weakest root development, barely visible root networks covering the soil, and the roots with the smallest fresh and dry weights despite being longer (Figure 3).

### 2.2. Morphological Characteristics of the Epidermal Cells and Stomata

**Figure 4:**
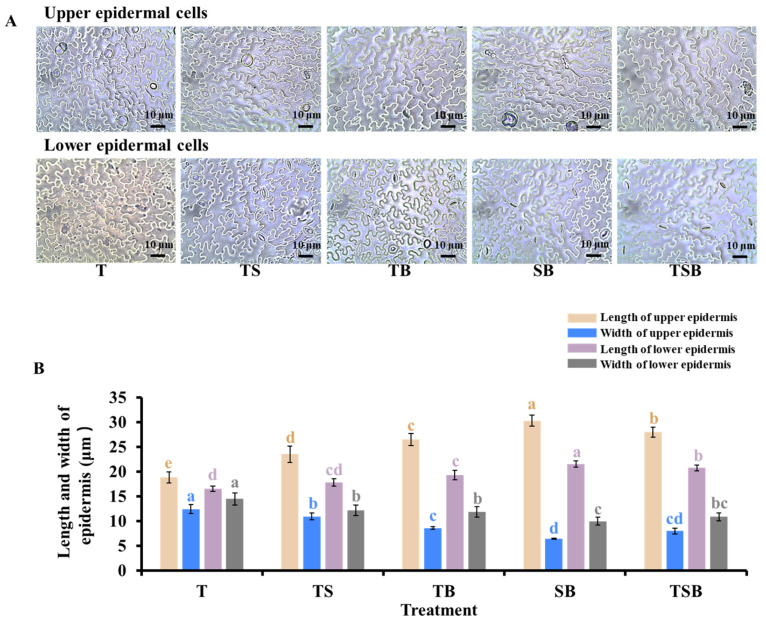
The trait of lettuce leaf epidermal cells under various lighting directions during 45 days of cultivation. Micrographs of epidermal cells (20×). (**A**) The micrograph of the upper and lower epidermal cells in the mature fourth leaf from the top; the bars indicate 10 μm. (**B**) The average length and width of upper and lower epidermal cells. Vertical bars represent means ± standard error (n = 9). Different lowercase letters indicate significant differences within treatments by Duncan’s multiple range test at *p* ≤ 0.05.

The lighting direction treatment considerably influenced the morphology in the epidermal cells of leaf midribs during their development (Figure 4). Lettuce leaves in the SB treatment were curled and grew to slant downward due to the light source from the side and bottom. The upper epidermal cells, which were pulled, and the lower epidermal cells, which were squeezed, were deformed and became the narrowest. In addition to the T treatment, lettuce leaves in other light direction combinations all grew in various directions due to different light source directions, resulting in different degrees of epidermal cell deformation, and the widest and shortest epidermal cells appeared in the T treatment.

**Figure 5:**
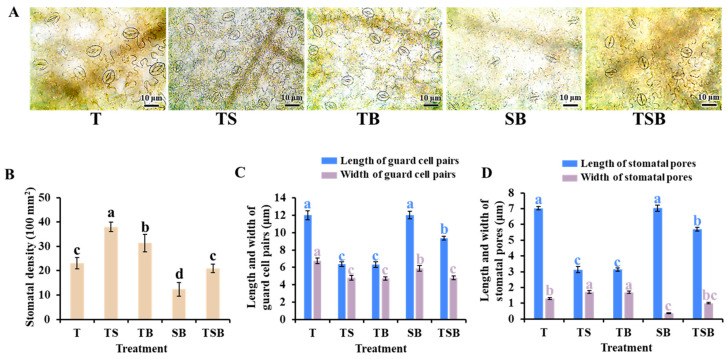
The trait of stomatal cells in the lower surface of lettuce leaves under various lighting directions during 45 days of cultivation. Micrographs of stomatal density and morphology (20×). (**A**) The micrograph of stomatal cells in the lower surface of the mature fourth leaf from the top; the bars indicate 10 μm. (**B**) The stomatal density per 100 mm^2^. (**C**) The length and width of guard cell pairs. (**D**) The length and width of stomatal pores. Vertical bars represent means ± standard error (n = 9). Different lowercase letters indicate significant differences within treatments by Duncan’s multiple range test at *p* ≤ 0.05.

As shown in Figure 5, the stomatal properties of lettuce leaves were highly affected by the lighting direction. In general, stomata in the leaves of terrestrial herbs are distributed in both the upper and lower epidermis, but mainly in the lower epidermis. In the treatment of different light directions, the lower epidermal cells were squeezed to varying degrees because the lettuce leaves tended to stretch in different directions. Generally, stomata and other epidermal cells were roughly located on the same plane, so the density and morphology of stomata were also affected to various degrees. For the conventional single top light source direction, the leaf growth was not interfered with by other light directions, so the stomatal density, shape, and opening degree on the lettuce leaves in the T treatment were in a general conventional state. Compared with the T treatment, the SB treatment significantly reduced the stomatal density in the lettuce leaves. In addition, the guard cells of stomata in the SB treatment became narrow and long, and the stomatal opening was minimal and almost closed. On the contrary, in the same observation area, the TS treatment significantly increased the stomatal density, the guard cell shape was short and round, and the stomatal opening was the largest. In addition, the TB treatment was also excellent in improving stomatal density and promoting stomatal opening.

### 2.3. Photosynthetic and Chlorophyll Fluorescence Characteristics

**Figure 6:**
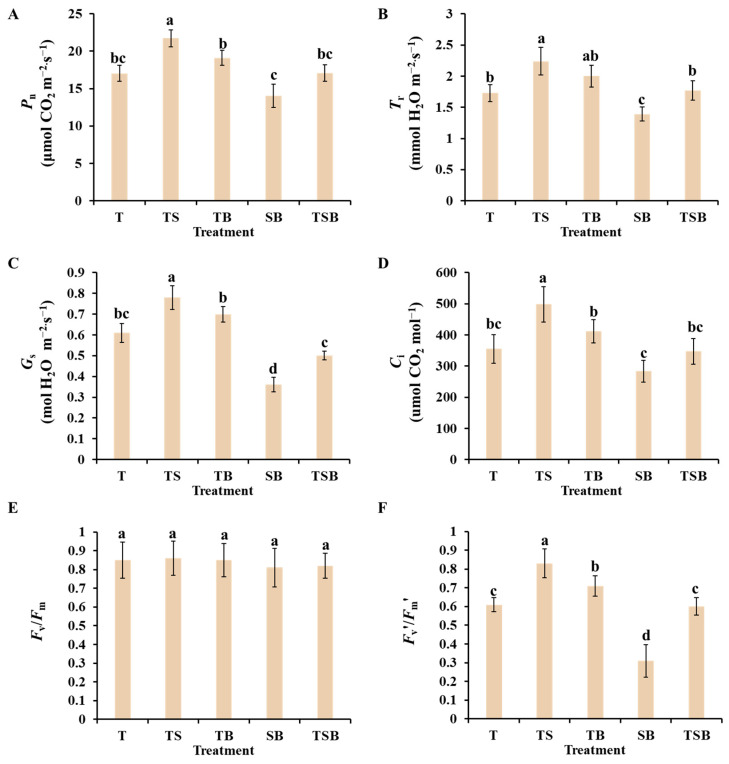
The photosynthetic and chlorophyll fluorescence characteristics of lettuce plants under various lighting directions during 45 days of cultivation. (**A**) Net photosynthetic rate. (**B**) Transpiration rate. (**C**) Stomatal conductance. (**D**) Intercellular CO_2_ concentration. (**E**) The maximal PSII quantum yield. (**F**) The photochemical efficiency of PSII. The above parameters of the mature leaves were measured by selecting from the top to the fourth round. Vertical bars represent means ± standard error (n = 9). Different lowercase letters indicate significant differences within treatments by Duncan’s multiple range test at *p* ≤ 0.05.

Figure 6A–D display the photosynthetic characteristics of lettuces under different lighting directions. The highest and lowest values of *P*_n_, *T*_r_, *G*_s_, and *C*_i_ were observed with TS and SB lighting, respectively. There was no significant difference in the *P*_n_, *T*_r_, *G*_s_, and *C*_i_ values of lettuce between the T and TSB treatments. These values were only slightly lower than those in the TB treatment. The increase in net photosynthetic rate shows that TS lighting is highly effective for photosynthesis. This may be due to the improvement of stomatal properties by the TS lighting.

Figure 6E,F display the chlorophyll fluorescence parameters *F*_v_/*F*_m_ and *F*_v_’/*F*_m_’ in response to different light directions. The *F*_v_/*F*_m_ remained stable in the non-stress environment. The *F*_v_/*F*_m_ remained consistent across all treatments. The highest *F*_v_’/*F*_m_’ was in the TS treatment, while the lowest was in the SB treatment. The TB treatment, although good, always had significantly lower values than the TS treatment. Furthermore, there was no difference in *F*_v_’/*F*_m_’ between treatments T and TSB, which was considerably lower than in the TB treatment. These findings indicate that the appropriate lighting direction combination, such as the TS treatment, is crucial for enhancing chlorophyll fluorescence characteristics and photosynthetic capacity in lettuce plants.

### 2.4. Carbohydrates and Soluble Proteins

**Figure 7:**
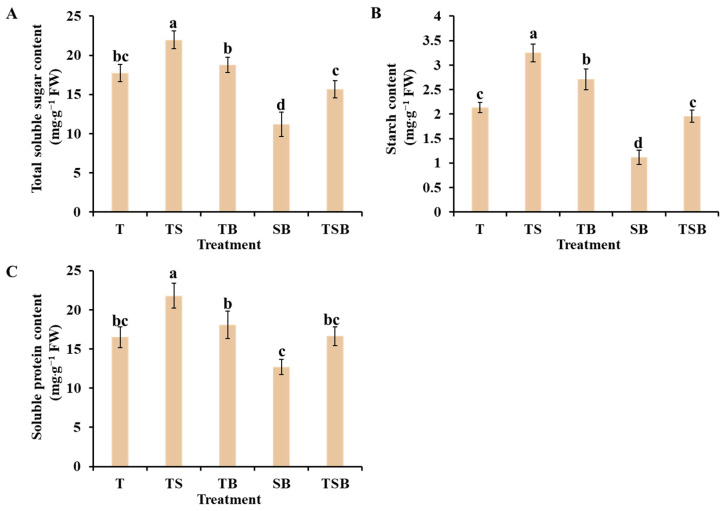
The content of carbohydrates and soluble proteins of lettuce plants under various lighting directions during 45 days of cultivation. (**A**) Total soluble sugar content. (**B**) Starch content. (**C**) Soluble protein content. The above parameters of the mature leaves were measured by selecting from the top to the fourth round. Vertical bars represent means ± standard error (n = 9). Different lowercase letters indicate significant differences within treatments by Duncan’s multiple range test at *p* ≤ 0.05.

The results above demonstrate that different combinations of lighting direction resulted in variations in photosynthetic efficiency (Figure 6). We explored how lighting directions impact the carbohydrate and soluble protein levels in lettuce leaves (Figure 7). The TS treatment had the largest carbohydrates and soluble proteins, while the SB treatment had the smallest. The TB treatment was also good but consistently lower than the TS treatment. Furthermore, there were no differences in carbohydrates and soluble proteins between treatments T and TSB, which were significantly lower than the TB treatment. In summary, the increased carbohydrates in lettuce leaves were affected by the movement of the leaves and adjustments in the lighting direction. These adjustments led to an increase in light absorption area and adaxial leaf petiole angle, resulting in higher photosynthetic efficiency and carbohydrate content in the plants [4]. Enhanced photosynthesis can elevate the overall metabolic level of plants. Most soluble proteins in plants function as enzymes involved in various metabolic processes, and their abundance serves as a crucial indicator of the plant’s total metabolic activity.

### 2.5. The Change in Chlorophyll Content, Chlorophyllase (CLH), and Pheophytinase (PPH) Activities

**Figure 8:**
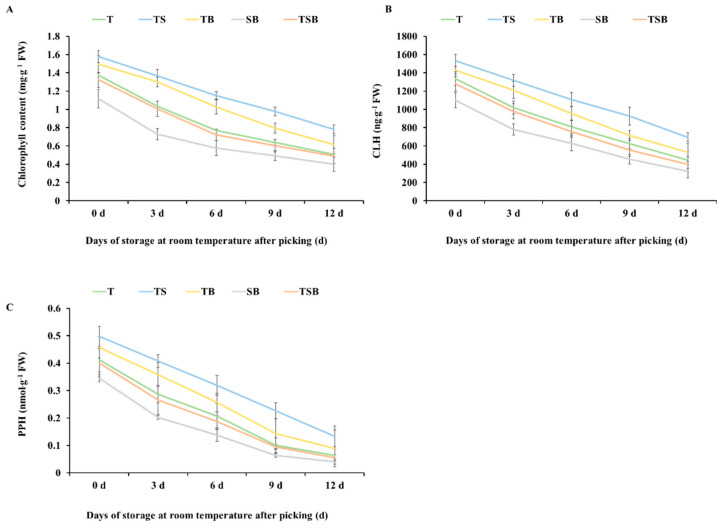
Cultured under various lighting directions for 45 days, after picking, the (**A**) chlorophyll content, (**B**) chlorophyllase (CLH), and (**C**) pheophytinase (PPH) contents in lettuce leaves during storage at room temperature (22 °C, 60% RH (Relative Humidity)) for 0, 3, 6, 9, and 12 days, respectively. The above parameters of the mature leaves were measured by selecting from the top to the fourth round. Vertical bars represent means ± standard error (n = 9).

The lettuce samples were collected after 45 days of lighting direction cultivation and placed at room temperature 22 °C and 60% RH for 0, 3, 6, 9, or 12 days with roots, respectively, and then the chlorophyll content and enzymatic activities of chlorophyllase (CLH) and pheophytinase (PPH) were determined (Figure 8).

Chlorophyll content is closely related to photosynthesis and nutritional status and is an essential indicator of plant growth. On the day of harvest, the chlorophyll content of lettuce grown in different light directions from high to low was TS, TB, T, TSB, and SB. Chlorophyll degradation is the most apparent sign of plant leaf aging [25]. Along with the extension of storage time after picking, the chlorophyll content of lettuce cultivated under different light treatments showed various degrees of reduction (Figure 8A). Compared with other treatments, the chlorophyll content of lettuce cultured under the TS treatment decreased gently from the day of harvest to the 12th day of storage at room temperature, from 1.576 to 0.782 mg·g^−1^ FW (fresh weight) and decreased by 50.38%. The chlorophyll content of lettuce cultured under the TB treatment was degraded rapidly from the 3rd day after storage and decreased by 58.88% from 1.493 to 0.614 mg·g^−1^ FW after storage at room temperature for 12 days. In the process of storage at room temperature after picking, the chlorophyll content of lettuce cultured under the T and TSB treatments yielded little difference, and the changing trend was similar. The chlorophyll content of lettuce cultured under the T treatment was degraded rapidly from the day of picking to the 6th day and gradually became stable from the 6th to the 12th day. Finally, the chlorophyll content of lettuce cultured under the T treatment was reduced from 1.373 to 0.507 mg·g^−1^ FW, and the TSB treatment decreased from 1.323 to 0.491 mg·g^−1^ FW and decreased by 63.07% and 62.89%, respectively. The chlorophyll content of lettuce cultured under the SB treatment degraded the fastest in the first three days after picking and storing at room temperature. Finally, the content decreased from 1.117 to 0.399 mg·g^−1^ FW, an overall decrease of 64.28%. It can be seen that the lettuces cultured under the TS treatment had the best storage effect at room temperature after picking, the chlorophyll degradation was the slowest in the same storage environment, and the lettuces cultured under the SB treatment had the worst performance.

The “post-harvest loss of green” will directly affect the sensory quality and shelf life of green leafy vegetables. Relevant studies have shown that CLH and PPH are critical enzymes in the chlorophyll degradation pathway [26,27]. As can be seen from Figure 8B,C, with the extension of storage time, the activities of CLH and PPH in lettuce cultured under any light direction were decreased, and the changing trend was similar to that of chlorophyll degradation. During storage at room temperature after picking, the activity of CLH in TS-treated lettuces decreased evenly to 694.643 ng·g^−1^ FW on day 12, decreasing by 40.63%; after 12 days of storage at room temperature, its content in SB-treated lettuces was decreased to 321.91 ng·g^−1^ FW, decreasing by 70.74%. The activity of CLH decreased to 529.28, 443.76, and 398.77 ng·g^−1^ FW in lettuce grown with the TB, T, and TSB treatments after 12 days of storage at room temperature, which decreased by 62.94%, 66.80%, and 68.81%, respectively. As for the change in PPH activity, after storage at room temperature until day 12, its content in TS-treated lettuce decreased to 0.133 nmol·g^−1^ FW, which decreased by 73.29%. The PPH activity in SB-treated lettuces decreased to 0.041 nmol·g^−1^ FW on the 12th day, which decreased by 88.12%. The PPH activity in lettuces cultivated by the TB, T, and TSB treatments decreased to 0.089, 0.064, and 0.055 nmol·g^−1^ FW at room temperature 12 days after picking, which was decreased by 80.57%, 84.47%, and 86.28%, respectively. It can be seen that the cultivation process of different light directions affects the physiological activities of lettuce and can regulate the expression level of enzymes related to the chlorophyll degradation pathway, thereby delaying chlorophyll degradation [28]. Moreover, the cultivation effect of the TS treatment on lettuce is better than that of other treatments.

### 2.6. The Change in Water Loss Rate

**Figure 9:**
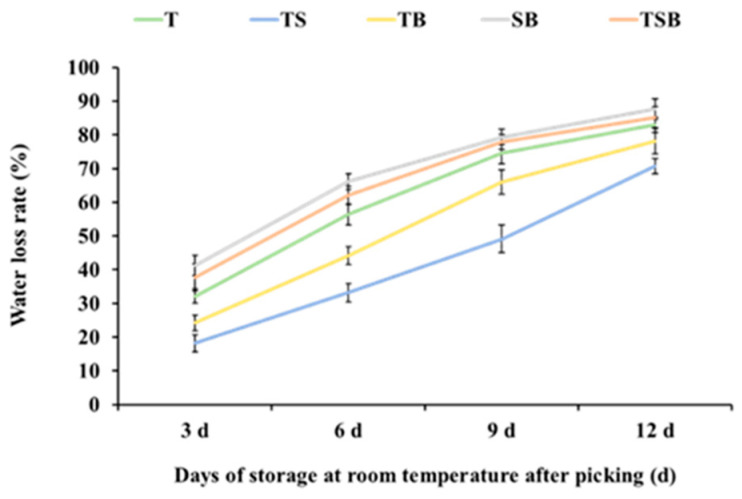
Cultured under various lighting directions for 45 days, after picking, the water loss rate in lettuce leaves during storage at room temperature (22 °C, 60% RH (Relative Humidity)) for 3, 6, 9, and 12 days, respectively. The above parameters of the mature leaves were measured by selecting from the top to the fourth round. Vertical bars represent means ± standard error (n = 9).

Water is an essential factor in keeping fruits and vegetables fresh, and the water content in fresh lettuce is very high, generally ranging from 90% to 95% [29]. With respiration and transpiration, fruits and vegetables continue to lose water after harvest, the weight continues to decline, and the leaves are damaged after cutting, more prone to water loss, the external performance is wilting, shriveling, and quality declines. As shown in Figure 9, lettuce cultivated under different light directions showed various degrees of water loss during storage at room temperature after picking. At room temperature, the lettuce cultured under the SB, TSB, and T treatments all lost water rapidly, and the samples were seriously wilted after 3 to 6 days of storage. The water loss rate was as high as 66.16%, 62.08%, and 56.37% on the 6th day, respectively. Still, the lettuce cultured under the TB and especially the TS treatments could effectively reduce the water loss rate; on the 6th day, the water loss rate was 44.17% and 33.17%, respectively, which yielded an excellent fresh-keeping effect. After picking and storing at room temperature for 9 days, the water loss rate of the lettuce cultured by the TS treatment increased rapidly, reaching 70.69% on the 12th day compared with the first harvest. The rapid water loss stage of lettuce cultured by the TB treatment was from 6 to 9 days after storage at normal temperature and gradually leveled off to 78.07% by the 12th day. On the 12th day of storage at room temperature, the water loss rate of T-, TSB-, and SB-cultured lettuce reached 82.99%, 85.21%, and 87.66%, respectively. The study of Li Xuerui et al. showed that the water loss rate of lettuce was as high as 40.03% on the 4th day of storage at room temperature [23], for which the data of the regular temperature control group were only collected after 4 days of storage, while the water loss rate of lettuces cultured under the TS treatment was only 33.17% on the 6th day of storage at room temperature. The above results show that differences in lettuce’s physiological structure, such as leaf thickness, leaf epidermal cell morphology, and stomatal morphology, are caused by different light directions during cultivation. These light directions jointly regulate lettuce’s water loss rate during post-harvest storage at room temperature. Compared with other treatments, the TS treatment can effectively prevent water loss for a certain period in the same storage environment.

### 2.7. The Change in Membrane Permeability

**Figure 10:**
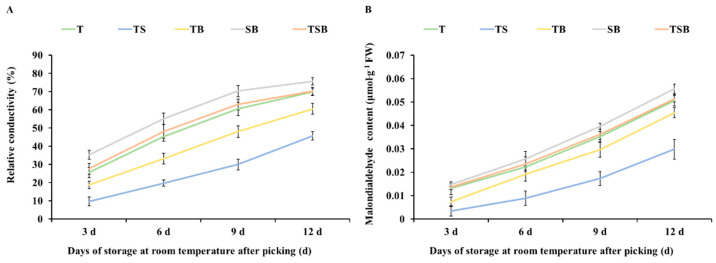
Cultured under various lighting directions for 45 days, after picking, the (**A**) relative conductivity and (**B**) malondialdehyde (MDA) content in lettuce leaves during storage at room temperature (22 °C, 60% RH (Relative Humidity)) for 3, 6, 9, and 12 days, respectively. The above parameters of the mature leaves were measured by selecting from the top to the fourth round. Vertical bars represent means ± standard error (n = 9).

The electrical conductivity can reflect the integrity of the cell membrane and the degree of senescence of the fruit. As can be seen from Figure 10A, during storage at room temperature after picking, the electrical conductivity of lettuce showed an increasing trend with the extension of storage time. Compared with SB-cultured lettuce, the TB and TS treatments could significantly mitigate the increase in electrical conductivity. Among them, the conductivity of lettuces cultured under the TS treatment increased the slowest, rising to 45.69% on the 12th day, followed by that under the TB treatment, rising to 60.47% on the 12th day. In comparison, the conductivity of lettuces cultured under the SB treatment was 75.66% on the 12th day, which was the highest among all treatments. It can be seen that lettuce cultures with the TS treatment have the best effect on alleviating the increase in conductivity and maintaining the integrity of the cell membrane.

The MDA content is regarded as one of the indexes used to judge the degree of aging [30]. As can be seen from Figure 10B, the MDA accumulation was apparent and rapid in lettuce cultured under the TB, T, TSB, and especially the SB treatment. When the lettuce was stored at room temperature for 3 days after picking, the MDA content in the sample was 0.0074, 0.0132, 0.0137, and 0.0148 μmol·g^−1^ FW. On the 12th day of storage, the MDA content was increased to 0.0455, 0.0507, 0.0514, and 0.0557 μmol·g^−1^ FW, respectively. The content of MDA in lettuce samples cultured under the TS treatment increased relatively slowly. After 3 days of storage, the MDA content was 0.0035 μmol·g^−1^ FW and increased to 0.0299 μmol·g^−1^ FW by day 12. Compared with other treatments, TS treatment could inhibit the accumulation of MDA, and the fresh-keeping effect was the best after storage at room temperature for a certain period.

### 2.8. The Change in β-Galactosidase (β-GAL) Activity and Brittleness

**Figure 11:**
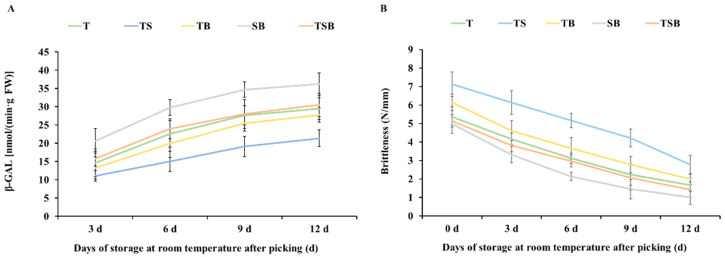
Cultured under various lighting directions for 45 days, after picking, the (**A**) β-Galactosidase (β-GAL) activity and (**B**) brittleness in lettuce leaves during storage at room temperature (22 °C, 60% RH (Relative Humidity)) for 0, 3, 6, 9, and 12 days, respectively. The above parameters of the mature leaves were measured by selecting from the top to the fourth round. Vertical bars represent means ± standard error (n = 9).

β-galactosidase (β-GAL) is one of the critical enzymes in cell wall degradation, which can catalyze the degradation of pectin polymers and destroy the cell wall structure, thus softening fruit, which is one of the essential manifestations of fruit and vegetable aging [31]. As shown in Figure 11, there was a negative correlation between brittleness and β-GAL activity. The greater the β-GAL activity, the faster the aging and softening speed of vegetable leaf cells will be, and the brittleness will be weakened.

As can be seen from Figure 11A, after picking and storing at room temperature for 3 days, the β-GAL activity in the lettuce samples cultured under SB treatment increased to 20.66 nmol·min^−1^·g^−1^ FW. The β-GAL activity in FW, TSB, T, TB, and TS was 15.79, 14.66, 13.29, and 11.07 nmol·min^−1^·g^−1^ FW, respectively. The activity of β-GAL in lettuce samples cultured under the SB treatment was almost twice that under the TS treatment. The activity of β-GAL in TS-treated lettuce increased slowly during storage, reaching 21.38 nmol·min^−1^·g^−1^ FW on day 12. The increased rate of β-GAL activity in other treatments slowed down after 6 days of storage. On the 12th day, the β-GAL activity in TB, T, TSB, and SB was 27.76, 29.48, 30.54, and 36.24 nmol·min^−1^·g^−1^ FW, respectively.

As shown in Figure 11B, there are differences in the brittleness of lettuce cultivated in different light directions. Lettuces cultivated under the TS treatment had the highest brittleness on the day of picking, followed by those treated with TB, with a brittleness of 7.12 and 6.14 N/mm, respectively. Lettuces treated with T and TSB had lower brittleness, and those treated with SB had the lowest, at 5.36, 5.14, and 4.97 N/mm, respectively. With the extension of room temperature storage time after picking, the brittleness of all lettuce samples decreased rapidly, and the brittleness of lettuce cultured under the TS treatment decreased slowly compared with other treatments. By the 12th day, the brittleness of lettuce samples was 2.78 (TS), 1.99 (TB), 1.66 (T), 1.43 (TSB), and 1.01 (SB) N/mm, respectively. Lettuces grown under the TS treatment were more than twice as brittle as those grown under the SB treatment. In this study, compared with other treatments, lettuces cultivated by the TS treatment could maintain brittleness better and slow down the aging rate. This is similar to the effect of alleviating the increase in conductivity and MDA accumulation.

## 3. Discussion

### 3.1. Variations in the Lighting Direction Combinations: Their Effects on Anatomical, Physiological, Morphological, and Growth Parameters of Lettuce Plants

Plants can adapt to changing environments, allowing for flexible growth and development [32]. In the current study, plants experienced significant effects in adapting to various lighting directions. For the lighting conditions affecting plant growth, when the combined light direction included side lighting, particularly from the bottom, it was observed that the upper epidermal cells in midribs were stimulated to a greater extent than the lower epidermal cells, leading to increased elongation (Figure 4). As a result, there was an increased leaf angle, and the leaves bent towards the light source to capture and utilize available light more efficiently (Figure 1). Thus, the SB treatment significantly reduced the plant height and canopy (Figure 1 and Table 1), leading to the development of long, narrow leaves. The highest fresh and dry weights of shoots were obtained in plants grown under TS lighting, which is consistent with the findings of a previous study. The study noted that TS lighting significantly improved fresh and dry weight, leaf number, area, and thickness in chrysanthemum ‘Pearl Egg’ and ‘Gaya Glory’ compared to those cultivated under single downward (overhead) lighting (Figure 2). The lettuces cultivated with TS lighting showed an increase in the number of leaves and larger leaf sizes, which can effectively capture and utilize available light. Interestingly, the lighting direction in this study significantly affected the leaf morphology and root biomass. Furthermore, compared to other treatments, the well-developed roots observed in lettuce plants under TS lighting may be enhanced by increased photosynthesis, which provides sufficient energy to the stems and roots (Figure 3) [33].

The morphological structures and physiological functions of plants are known to be closely interrelated. The morphology of leaf structures is closely related to variations in photosynthetic rate. The lettuce plants exhibited the most well-developed leaf anatomical structures in response to TS lighting. The epidermal morphology can be affected by changes in lighting direction [5,6,34]. Plant shoots, particularly leaves, tend to bend towards light to capture and utilize available light effectively. This behavior is attributed to positive phototropism [35,36]. When lettuce plants are exposed to light from various angles, especially oblique ones, the angle of the adaxial leaf petiole changes, causing the leaves to bend towards the light (Figure 2). The upper epidermal cells in the midribs were prolate, while the lower epidermis was constricted, resulting in wide and flat cells (Figure 4). Stomata are pores for gas exchange in the epidermis of plant leaves, stems, and other organs. They are closely associated with photosynthesis. In our study, the movement of leaves induced by phototropism led to changes in leaf angle, which then stimulated epidermal cells and influenced the stomatal state. Figure 5 shows that lettuce plants grown under TS lighting had the highest stomatal density, with open pores and smaller sizes. As a result, the superior stomatal characteristics demonstrate enhanced photosynthetic efficiency and robust stress resistance in lettuces cultivated under TS lighting [12,37,38].

The application of TS lighting in this study significantly increased the levels of *P*_n_, *T*_r_, *G*_s_, and *C*_i_ in lettuces (Figure 6A–D). Enhanced photosynthetic characteristics resulted in an increase in carbon gain and the growth of lettuce [39]. Additionally, the well-developed leaf structures, high chlorophyll content (Figure 8A), and increased density of open stomata were closely associated with the improved net photosynthetic rate in lettuces under TS lighting conditions [40,41,42].

Higher electron flow through PSII is consistently associated with improved photosynthetic capacity [43]. Chlorophyll fluorescence properties play a crucial role in regulating photosynthesis and plant responses to environmental factors due to their sensitivity and convenience [44]. The fluorescence of chlorophyll is closely related to many photosynthetic processes. The impact of stress on photosynthesis can be seen in the fluorescence kinetics of chlorophyllin [45]. Prior research has shown a strong positive relationship between fluorescence characteristics and chlorophyll concentration in living plant leaves [46]. This study found that lettuce plants showed significant improvements in the photochemical efficiency of PSII (*F*_v_′/*F*_m_′) in response to TS lighting (Figure 6F). These findings show that the optimal lighting direction can improve PSII efficiency and potentially boost photosynthesis by facilitating energy transfer from PSII to PSI.

Additionally, the lighting direction variations in lettuce plants impacted the accumulation of primary metabolites. Carbohydrates, like starch and soluble sugars, are the direct products of efficient photosynthesis and are essential for plant growth and development [47]. The soluble protein content is a crucial indicator of plant metabolism, serving as a remarkable physiological and biochemical index. The TS lighting significantly increased carbohydrate and soluble protein levels (Figure 7) due to excellent stomatal characteristics, high chlorophyll concentrations, and efficient light utilization. Moreover, Feng et al. indicated that the sucrose, starch, and total soluble sugar content in soybean plants were significantly increased with increasing light intensity [48]. Additionally, according to Min et al., different End of Production (EoP) light intensities also affected the level of total carbohydrates (glucose + fructose + sucrose + starch) in lettuce [24]. Carbohydrate levels at the end of EoP light treatments were linearly correlated with applied light intensities in their study. Glucose, sucrose, fructose, and starch levels showed similar responses to light intensity as the total carbohydrates level. Overall, the optimum light intensity and direction, which positively changed the leaf orientation and adjusted the leaf angle to perpendicular to coming light, improved enzymatic activities related to sucrose and starch, and increased the accumulation of primary metabolites; consequently, plants grow well under prevailing conditions [5,6,24,48,49].

### 3.2. Variations in the Lighting Direction Combinations: Relationship between Culture Environmental Lighting and Post-Harvest Fresh-Keeping of Lettuce Plants

To achieve optimal growth and development, plants rely heavily on light as an essential environmental cue. The diverse responses of plants to light necessitate a sophisticated sensing of its intensity, direction, duration, and wavelength [50]. Compared to the top light culture environment, adding side light enhanced the development of leaf tissue structures. As illustrated in Figure 2, the TS light led to the growth of the thickest lettuce leaves. Some studies have reported that TS lighting significantly increased leaf thickness and improved the development of palisade and spongy tissues. Compared to SB lighting, TS light developed well-defined leaves with clear and compact structures [4,5,6,51]. While water is primarily lost in plants through transpiration, this process occurs in leaves in two different ways: (1) Transpiration through the stratum corneum is known as corneum transpiration [52]. A thick leaf is a morphological feature of a plant leaf, typically characterized by an increased number of cell layers and more stomata. The leaves have been adapted to withstand harsh environmental conditions like drought and high temperatures. This adaptation helps the plants conserve water more effectively and facilitate photosynthesis [52]. As illustrated in Figure 5, the lettuce leaves cultured with the TS treatment exhibited dense and small stomata. (2) Transpiration through stomata, known as stomatal transpiration, is considered the primary mode of plant transpiration [52]. Stomata are an essential channel for gas and water exchange between leaves and the external environment, and they play a crucial role in plant photosynthesis and transpiration. When exposed to environmental stress, a plant will exhibit various responses to alleviate the stress and enhance its resistance. For instance, by detecting environmental and internal signals through guard cells, plants can regulate the transportation of water and photosynthesis to maintain a balance. This enables them to sustain a certain photosynthetic capacity without excessive water loss [53]. In accordance with Figure 6, lettuces subjected to the TS treatment exhibited increased transpiration and net photosynthetic rates. Normal transpiration promotes CO_2_ assimilation as it results in stomata opening during leaf transpiration, creating a pathway for CO_2_ to enter the leaf [52]. In addition, soluble protein is a crucial osmotic regulator and nutrient, and its increase and accumulation can improve the water retention capacity of cells and play a protective role in the membrane (Figure 7C) [54].

Traits associated with “green holding” can extend the crop’s ability to maintain photosynthetic capacity during reproductive growth, increasing biomass accumulation and improving crop yield [55]. Figure 8A illustrates that lettuce subjected to the TS treatment exhibited the highest chlorophyll content on the day of harvest. With the extension of post-harvest storage time, the rate of chlorophyll degradation was found to be the slowest among all treatments, thereby maintaining the “green holding” traits of leaves for an extended period. All factors that affect chlorophyll metabolism will lead to the loss of green in plants. The possible reasons for this phenomenon are as follows: (1) Water scarcity has been shown to affect chlorophyll biosynthesis and may lead to accelerated decomposition of the original chlorophyll [56]. In this study, the lettuce cultivated using the TS treatment exhibited thicker leaves and a higher stomatal density, which contributes to the regulation and maintenance of leaf water content more efficiently. (2) The process of chlorophyll biosynthesis involves a series of enzymatic reactions influenced by leaf temperature [56]. Transpiration can reduce plants’ temperatures, resulting in a significant loss of radiant heat [57]. (3) Nutrient deficiency can lead to the loss of green color in plants [56]. The direction of TS light significantly enhanced the accumulation of organic nutrition in lettuces, resulting in improved nutrition and reduced aging (Figure 7).

This study demonstrated significant variations in the growth state, leaf anatomical structure, photosynthetic coefficient, and nutritional composition of lettuce when cultivated under different lighting directions. The post-harvest storage stage of plants can be likened to an environment of “water and nutrient deprivation,” during which the freshness of plant products can only be prolonged through the reasonable allocation of their inherent water and nutrition. The application of the TS treatment increased the thickness of leaves and a higher density of stomata. This indicates a potential capacity to regulate water loss and somewhat enhance photosynthetic activity. During the post-harvest storage period, the leaves demonstrated positive water retention, contributing to a slowdown in chlorophyll degradation. Effective management of the “wilting and yellowing” of plant leaves is the key to keeping the leaves fresh and green. Low water loss rate (Figure 9), low membrane permeability (Figure 10), high brittleness (Figure 11), and long green retention duration of leaves are the characteristics of anti-aging in post-harvest storage of green leafy vegetables. These characteristics contribute to preserving quality and freshness in leafy vegetables during storage, which is vital for their shelf life and consumer appeal.

## 4. Materials and Methods

### 4.1. Plant Materials, Growth Conditions, and Experimental Setup

**Figure 12:**
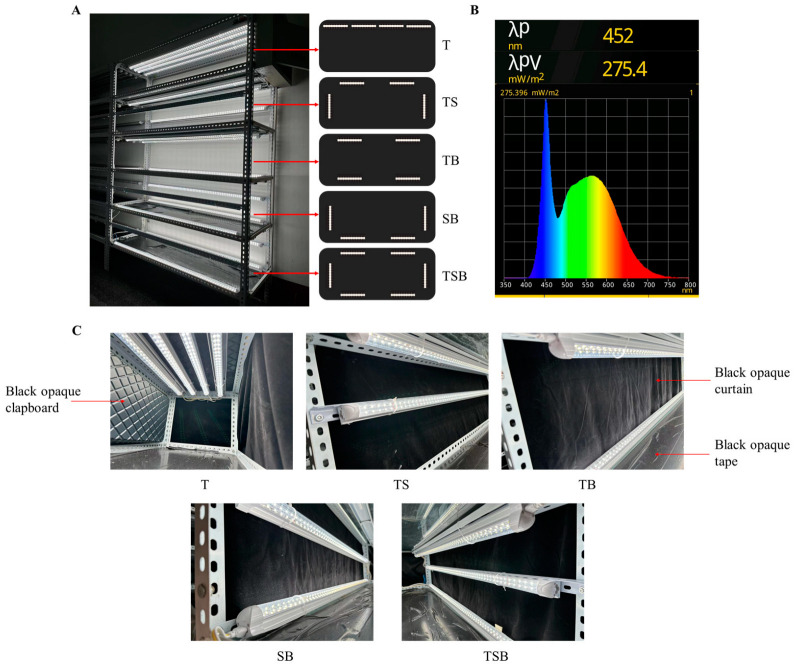
The experimental layout and design of the lighting direction combinations. (**A**) One of the plant culture shelves in the closed-type plant factory. The T, TS, TB, SB, and TSB refer to the top (1/1), top (1/2) + side (1/2), top (1/2) + bottom (1/2), side (1/2) + bottom (1/2), and top (1/3) + side (1/3) + bottom (1/3) lighting, respectively; please see the detailed information in Table 2. (**B**) The experimental light treatments utilized white LEDs with a spectral distribution of ~400–750 nm, peaking at 452 nm. The light period followed a 12 h day/night cycle starting at 8:00 a.m. daily. (**C**) Shading treatments between layers treated with different light directions.

One lettuce cultivar, ‘Caesar Green’ (Asia Seed Co., Ltd., Seoul, Republic of Korea), was chosen for the examination of the phenotypic responses to the lighting direction. The seeds were planted in 72-cell trays (72-Zigpot/72-cell tray, Daeseung, Jeonju, Republic of Korea) with commercial BVB medium (Bas Van Buuren Substrates, EN-12580, De Lier, The Netherlands) for germination on 3 March 2022. After observing “the first group of true leaves unfolded” of lettuce seedlings, they were transplanted into 10 × 10 cm plastic pots (Daeseung, Jeonju, Republic of Korea) filled with BVB medium. After transplanting, there were three plant culture shelves in a closed-type plant factory (770.0 cm long by 250.0 cm wide by 269.5 cm high, Green Industry Co., Ltd., Changwon, Republic of Korea) that were used for the light treatments at day 20 °C/night 10 °C and a 75% relative humidity (RH). An online CO_2_ sensor (Model No. GMT220 Carbocap, Vaisala, Vantaa, Finland) monitored the CO_2_ concentration of 1100–1200 ppm from a compressed gas tank to support plant photosynthesis. Air circulated horizontally through evenly distributed apertures in the cultivation rooms. The transplanted seedlings were randomly divided into 15 groups (each group contained nine plants) and transferred into these three separate plant culture shelves. As shown in Figure 12A, each shelf was equally divided into five compartments according to the combination of lighting directions. To prevent light interference, all reflective areas on the shelves and plates of each layer were covered with an opaque black curtain, clapboard, or tape (Figure 12C). Every plate contained one group of plants, with 10 cm intervals between each plant. The three plant culture shelves were used as three repetitions with the same setup.

The custom LED light (SungKwang LED Co., Ltd., Incheon, Republic of Korea) has a wide spectrum ranging from 400 to 750 nm with a peak at 452 nm in blue (Figure 12B) and was used from 08:00 to 20:00 daily. It was determined using a hand-held spectroradiometer (MK550T, UPRtek, Miaoli, Taiwan, China). The PWM (pulse width control method) LED dimmer was used in different directions to maintain consistent light intensity in each treatment to ensure that lettuce plants were exposed to the light from various directions for a sum of light intensity of 600 μmol·m^−2^·s^−1^ PPFD (Table 2). Furthermore, LED lamps were positioned 10 cm away from the plants in different directions. The light intensity was measured using a quantum radiation probe (FLA 623 PS, ALMEMO, Holzkirchen, Germany) at the top-leaf level of the plant.

The plants were watered daily at 9:00 a.m. with a nutrient solution composed of (in mg∙L^−1^) 708.0 Ca(NO_3_)_2_∙4H_2_O, 246.0 MgSO_4_∙7H_2_O, 505.0 KNO_3_, 230.0 NH_4_H_2_PO_4_, 1.24 H_3_BO_3_, 0.12 CuSO_4_∙5H_2_O, 4.00 Fe-ethylene diamine tetraacetic acid, 2.20 MnSO_4_∙4H_2_O, 0.08 H_2_MoO_4_, and 1.15 ZnSO_4_∙7H_2_O. Additionally, our study was not only designed as a completely randomized layout but also had 27 biological replications per treatment with consistent growth to minimize external influences.

### 4.2. Measurements of Morphological, Growth Parameters, and Post-Harvest Storage Condition

The growth parameters were measured, and the plants were harvested after 45 days of cultivation. They were then placed in liquid N_2_ in a −80 °C refrigerator for physiological analyses. To measure growth parameters, entire plants were harvested, and roots were carefully washed with tap water before being cut from the shoot. The shoot height, crown width, and fresh weight, as well as the number, length, and width of leaves, as well as root length and fresh weight, were directly measured. The shoots and roots were dried for five days at 65 °C in a Venticell-222 dry oven (MMM Medcenter Einrichtungen GmbH., Munich, Germany) before measuring their dry weights. For the lettuce post-harvest freshness test, the samples were collected after 45 days of cultivation and placed in the room (temperature 22 °C, 60% RH, about mid-April 2022) for 0, 3, 6, 9, or 12 days with roots, respectively. Then, the relevant indicators (water loss rate, relative conductivity, malondialdehyde (MDA) content, brittleness, chlorophyll content, enzymatic activities of β-Galactosidase (β-GAL), chlorophyllase (CLH), and pheophytinase (PPH)) were determined.

### 4.3. Anatomical Observation of Leaves

**Figure 13:**
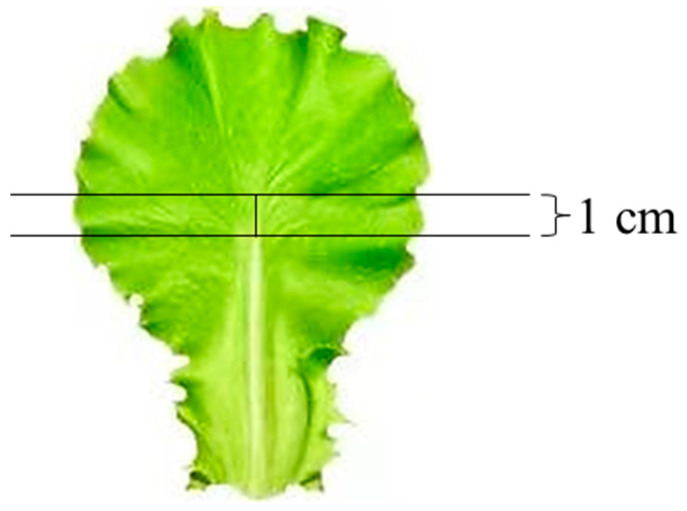
Freehand slice cutting range (the middle section of the mature fourth leaf from the top of the treated plants).

After 45 days of cultivation, for each lighting direction treatment, nine leaf segments (1 cm^2^) were collected from the middle section of the mature fourth leaf from the top of the treated plants (Figure 13). The tissue segments were immersed in a formaldehyde solution containing 5% (*v/v*) formalin, 5% (*v*/*v*) acetic acid, and 90% (*v*/*v*) ethanol at 4 °C for three days. The leaf samples were dehydrated three times in a graded series of ethanol solutions (95%, 75%, 50%, 25%, and 10% *v*/*v*) for each treatment, with each dehydration lasting for 40 min. After dehydration, the samples were sliced to an appropriate thickness using the freehand slice method. The slices were placed on glass slides and observed without staining using an optical microscope (ECLIPSE Ci-L, Nikon Corporation, Tokyo, Japan). ImageJ (ImageJ 1.48v, NIH, Bethesda, MD, USA) was used to measure the leaf thickness.

The upper and lower epidermis of midrib-less leaves were carefully removed from fully expanded leaves at a similar position to observe their epidermal and stomatal morphology. The stomata were observed by removing the epidermis from the leaf using gummed tape [58]. The stomatal traits were directly observed in fixed and discolored leaf samples. At 9:00 a.m., one hour after the beginning of daily photoperiodic treatments, the highest stomatal opening rates were typically observed, attributed to the peak photoperiodic activity. The fourth mature leaf at the top of the treated plant was selected from a single plant as a biological replicate, and three technical replicates and three biological replicates were performed for each experiment. The excised leaf circular segments (diameter = 1 cm) were fixed at 4 °C for 24~48 h in the formalin-acetic acid-alcohol (FAA) solution, which included 50% (*v*/*v*) ethanol, 45% (*v*/*v*) paraformaldehyde, and 5% (*v*/*v*) glacial acetic acid. Secondly, dehydration was carried out in a graded series of ethanol solutions (95%, 75%, 50%, 25%, and 10% *v*/*v*), with each solution being applied for 15 min, three times in total. Thirdly, decolorization was performed in a mixed solution containing 45% (*v*/*v*) ethanol, 45% (*v*/*v*) acetone, and 10% (*v*/*v*) distilled water at 4 °C for incubation lasting between 24 and 48 h. Finally, the treated sample slices were mounted on glass slides, and the leaf abaxial side was observed using an optical microscope (ECLIPSE Ci-L, Nikon Corporation, Tokyo, Japan) (micrographs of epidermal cells, stomatal density, length, and width of stomatal pores, magnification 20×), and analyzed with ImageJ (ImageJ 1.48v, NIH, Bethesda, Maryland, USA). Furthermore, the stomatal density was assessed following the description provided by Sack and Buckley [11]. In contrast, the dimensions of stomatal pores were determined in accordance with the methodology outlined by Chen et al. [59].

### 4.4. Measurements of Photosynthetic and Chlorophyll Fluorescence Indexes

On the last day of the treatment duration, before the harvest, the Li-6400 portable photosynthesis system (LI-COR Inc., Lincoln, NE, USA) was utilized to measure the photosynthetic parameters of the mature fourth leaf from the top of the treated plants. All parameters, including the net photosynthetic rate (*P*_n_), transpiration rate (*T*_r_), stomatal conductance (*G*_s_), and intercellular CO_2_ concentration (*C*_i_), were measured from 9:00 to 11:00 in a closed-type factory under a steady light intensity of 600 μmol·m^−2^·s^−1^ PPFD, environmental temperature of 20 °C, 75% RH, and CO_2_ concentration of 1100–1200 ppm.

We used a photosystem (Fluor Pen FP 100, Photon Systems Instruments, PSI, Drásov, Czech Republic) to measure chlorophyll fluorescence in lettuce leaves. Leaves were dark-adapted with a leaf clip for 30 min, then given a saturating light pulse of 0.6 s (3450 μmol·m^−2^·s^−1^ PPFD) to measure maximum (*F*_m_) and minimum (*F*_0_) fluorescence. The leaves were light-adapted for 5 min with continuous actinic light at 600 μmol·m^−2^·s^−1^ PPFD, similar to the growth condition, with saturating pulses every 25 s. Then, the maximum light-adapted fluorescence (*F*_m_′) and steady-state fluorescence (*F*_s_) were recorded. The *F*_v_/*F*_m_ was calculated as (*F*_m_ − *F*_0_)/*F*_m_ [60]. After excitation with PSI (*F*_0_′), the actinic light was turned off, and a far-red pulse was used to achieve minimal fluorescence. The *F*_v_′/*F*_m_′ = (*F*_m_′ − *F*_s_)/*F*_m_′ equation was then employed to calculate the *F*_v_′/*F*_m_′.

### 4.5. Measurements of Carbohydrates and Soluble Proteins

After 45 days of growth, leaves at the same stage were collected for carbohydrate measurements either at the end of the day or at night. The Anthrone colorimetric method by Vasseur and Ren et al. was used to measure starch and soluble sugars [61,62]. The technique for extracting soluble proteins is as follows: Fresh leaves were collected and immediately immersed in liquid nitrogen. They were then ground into a fine powder over an ice bath. An amount of 100 mg of the powder was homogenized in 50 mM of PBS (1 mM EDTA, 1 mM polyvinylpyrrolidone, and 0.05% (*v*/*v*) triton-X, pH = 7.0). The resulting mixture was centrifuged at 13,000 rpm and 4 °C for 20 min to obtain the supernatant, which would be used for the total protein estimation and enzyme activity assay [63]. The total protein levels were determined using Bradford’s reagent [64,65], while the contents of carbohydrates and soluble proteins were measured using a UV spectrophotometer (Libra S22, Biochrom Ltd., Cambridge, UK).

### 4.6. Measurement of Water Loss Rate

The samples were collected after 45 days of growth and placed in the room (temperature 22 °C, 60% RH, about mid-April 2022) for 3, 6, 9, or 12 days with roots, respectively. Then, the water loss rate was calculated using the following formula:

Water loss rate = (m_0_ − m)/m_0_ × 100%

m_0_: the quality of the samples before storage (g);

m: the quality of the samples after storage (g).

### 4.7. Measurement of Relative Conductivity

After harvesting, the plant samples were taken after 3, 6, 9, and 12 days of storage in room conditions (22 °C, 60% RH, about mid-April 2022) to determine the relative conductivity. The sample was made into tissue discs of uniform thickness and size with a perforator. An amount of 2 g was accurately weighed and placed in a beaker containing 20 mL distilled water and soaked for 1 h after oscillation to determine the relative conductivity of the extract solution C_1_. After the determination, it was boiled for 5 min, cooled, and supplemented with distilled water to 20 mL, and the relative conductivity C_0_ was determined. The following formula was used to calculate the relative conductivity.

The relative conductivity = C_1_/C_0_ × 100%

### 4.8. Measurement of Malondialdehyde (MDA) Content

After harvested, the plant samples were taken after 3, 6, 9, and 12 days of storage at room condition (22 °C, 60% RH, about mid-April 2022) to determine the malondialdehyde (MDA) content. An amount of 2 g of the sample were weighed. Then, 5 mL of trichloroacetic acid was added. The mixture was ground and centrifuged in an ice bath, and the resulting supernatant was taken as the MDA extract. Next, 2 mL of the MDA extract was mixed with 2 mL of 2% thiobarbituric acid and then placed in a water bath at 100 °C for 30 min. After cooling, the UV spectrophotometer (Libra S22, Biochrom Ltd., Cambridge, UK) was used to monitor the enhancement of A450 nm, A532 nm, and A600 nm. The molality concentration of MDA was then calculated using the formula below.

*C* = 6.45 × (A_532_ − A_600_) − 0.56 × A_450_

### 4.9. Measurement of Brittleness

After harvesting, the plant samples were taken after 0, 3, 6, 9, and 12 days of storage in room condition (22 °C, 60% RH, about mid-April 2022) to determine brittleness. The brittleness of lettuce leaf was measured using a textometer (TA. X TC-16, Baosheng Technology, Shanghai, China) in a single-cut mode. The maximum shear force (N) and maximum displacement (mm) were measured, and the slope of the curve was used to represent its brittleness; that is, the slope (brittleness) = the maximum shear force/maximum displacement, the larger the slope, the greater the hardness or brittleness of the sample, otherwise the smaller. The determination parameters were set as 0.1 N starting force, 40 mm/s test speed, and 10 mm return distance.

### 4.10. Measurement of Chlorophyll Content

The plant samples were harvested and stored at room condition (22 °C, 60% RH) for 0, 3, 6, 9, and 12 days in mid-April 2022. Chlorophyll content was determined using Arnon’s method with minor modifications [66]. In short, 0.2 g of fresh plant leaves were submerged in 2 mL of a mixture medium (45% *v*/*v* ethanol, 45% *v*/*v* acetone, 10% *v*/*v* distilled water) and incubated overnight at 4 °C with mild shaking using a rotator (AG, FINEPCR, Seoul, Republic of Korea). Subsequently, the supernatant was transferred to a cuvette and absorbance was measured at 645 nm and 663 nm by a spectrophotometer (Libra S22, Biochrom, Cambridge, UK). The total chlorophyll content was quantified individually using the following formula:

Total chlorophyll content (mg/g) = (20.20 × A_645_ + 8.02 × A_663_) × V/(100 × W)

V: the volume of the extraction mixture solution used;

W: the sample fresh weight.

### 4.11. Measurements of Activities of β-Galactosidase (β-GAL), Chlorophyllase (CLH), and Pheophytinase (PPH)

After harvested, the plant samples were taken after 0, 3, 6, 9, or 12 days of storage at room condition (22 °C, 60% RH, about mid-April 2022) to determine the enzymatic activities of β-GAL, CLH, and PPH. A 0.1 g fresh sample of lettuce leaves was taken, and the extract was ground in an ice bath. After centrifugation, the supernatant was obtained by using a β-GAL activity assay kit (Suzhou Kaming Biotechnology Co., Ltd., Suzhou, China), CLH content assay kit (Shanghai Enzyme Linked Biotechnology Co., Ltd., Shanghai, China), and PPH content assay kit (Shanghai Enzyme Linked Biotechnology Co., Ltd.) instruction manual to determine the activities of these three enzymes. The absorbance at 400 nm was determined, and the production of 1 nmol p-nitrophenol per gram of tissue per minute was defined as one β-GAL activity unit. Absorbance values were measured at 450 nm wavelength, respectively, according to the standard curve y = 0.0086x + 0.0532 (R^2^ = 0.9988; x: the concentration; y: the absorbance value) to calculate the CLH content in the plant samples, and according to the standard curve y = 0.0199x + 0.2459 (R^2^ = 0.9994; x: the concentration; y: the absorbance value) to calculate the PPH content.

### 4.12. Statistical Analysis

The plants were randomly sampled for this study, and data processing, plotting, and statistical analysis were conducted using Excel 2016 and the DPS package (DPS for Windows, 2009). ANOVA was used to analyze treatment differences, followed by Duncan’s multiple range test at a significance level of *p* ≤ 0.05 using SAS V. 9.1 (Statistical Analysis System, Cary, NC, USA). We used Student’s t-test to compare the differences between each treatment (*p* ≤ 0.05). Nine biological replicates were conducted to obtain all results, including measurements, calculations, and observations, which are presented as mean ± standard error.

## 5. Conclusions

This study pertains to plant cultivation, specifically focusing on promoting the growth and extension of post-harvest freshness for leafy vegetables. In this study, the combination of “top + side” lighting with a total light intensity of 600 μmol·m^−2^·s^−1^ PPFD has been employed to decrease shoot height, increase crown width, and length, width, number, and thickness of leaves and enhance the chlorophyll fluorescence characteristics, photosynthetic capacity, and nutrient content of lettuce. In particular, leaf thickness, stomatal density, and soluble protein content of lettuce significantly increased in TS treatment. Reasonable regulation of post-harvest water loss in lettuce is crucial for prolonging original chlorophyll stability and quantity. It was utilized to mitigate the increase in conductivity, suppress the accumulation of MDA, decrease the activity of β-GAL, and maintain the brittleness in lettuce, thereby decelerating their aging process. Overall, TS treated-lettuce performed best in controlling water loss, keeping visual value, and extending shelf life during storage after picking. This study also introduces a novel idea regarding the relationship within the cultivation light environment, the improvement of morphophysiological characteristics, and the extension of post-harvest freshness.

## Figures and Tables

**Table 1:** The influence of different lighting direction combinations on traits of lettuce shoots and leaves for 45 days of cultivation.

Treatment	Shoot				Leaf ^4^		
	Height ^2^ (cm)	Crown Width ^3^ (cm)	Fresh Weight (g)	Dry Weight (g)	Number	Length (cm)	Width (cm)
T	19.87 ± 1.76 b ^1^	28.67 ± 1.27 b	61.14 ± 2.34 c	6.67 ± 0.96 c	15.67 ± 1.93 b	13.96 ± 1.12 c	11.63 ± 0.91 ab
TS	17.73 ± 2.03 c	31.07 ± 1.54 a	83.10 ± 2.77 a	15.13 ± 1.06 a	21.33 ± 1.37 a	14.86 ± 1.33 bc	12.43 ± 0.82 a
TB	23.70 ± 2.17 a	25.40 ± 2.01 c	72.40 ± 2.15 b	10.53 ± 1.11 b	16.03 ± 1.88 b	15.90 ± 1.23 b	11.03 ± 0.67 b
SB	8.77 ± 1.86 d	19.23 ± 1.66 d	27.69 ± 1.78 d	2.36 ± 0.99 d	12.06 ± 1.11 c	18.90 ± 1.93 a	8.47 ± 0.94 c
TSB	18.07 ± 1.79 bc	25.65 ± 1.91 c	60.91 ± 2.11 c	5.62 ± 1.02 cd	15.73 ± 1.96 b	17.00 ± 1.90 ab	10.30 ± 0.72 bc

^1^ Different lowercase letters indicate significant differences within treatments by Duncan’s multiple range test at *p* ≤ 0.05, and the values are average ± standard error (n = 9). ^2^ The shoot height was measured as the height of the aboveground part of the plant. ^3^ The top view crown width. ^4^ The leaves with a length > 1 cm were counted to determine the total number of leaves per plant. The length and width of the mature leaves were measured by selecting from the top to the fourth round.

**Table 2:** Light intensity design of the lighting direction combinations.

Treatment	Abbreviation	Light Intensity (μmol∙m^−2^∙s^−1^ Photosynthetic Photon Flux Density (PPFD)) per Light Direction ^1^
Top (1/1)	T	600
Top (1/2) + Side (1/2)	TS	300
Top (1/2) + Bottom (1/2)	TB	300
Side (1/2) + Bottom (1/2)	SB	300
Top (1/3) + Side (1/3) + Bottom (1/3)	TSB	200

^1^ To ensure that lettuce plants were exposed to the light from different directions for a sum of light intensity of 600 μmol·m^−2^·s^−1^ PPFD.

## Data Availability

Data are contained within the article.
